# Identification of PIWI-interacting RNAs based models for lung adenocarcinoma early detection: a multicenter cohort study

**DOI:** 10.1186/s43556-025-00368-2

**Published:** 2025-11-30

**Authors:** Shuang Liang, Qian Hong, Qingxia Xu, Yuan Wang, Yue Wu, Juwei Mu, Chunyan Wang, Hezhi Fang, Wei Cui

**Affiliations:** 1 Department of Clinical Laboratory, State Key Laboratory of Molecular Oncology, National Cancer Center/National Clinical Research Center for Cancer/Cancer Hospital, Chinese Academy of Medical Sciences and Peking UnionMedical College, Beijing, 100021 China; 2 Department of Thoracic Surgery, National Cancer Center/National Clinical Research Center for Cancer/Cancer Hospital, Chinese Academy of Medical Sciences and Peking Union Medical College, Beijing, 100021 China; 3 Department of Thoracic Surgery, Sir Run Run Shaw Hospital, School of Medicine, Zhejiang University, Hangzhou, Zhejiang 310058 China; 4 Department of Clinical Laboratory, Affiliated Cancer Hospital of Zhengzhou University & Henan Cancer Hospital, Zhengzhou, Henan China; 5 Department of Transfusion, Shanxi Province Cancer Hospital/Shanxi Hospital Affiliated to Cancer Hospital, Chinese Academy of Medical Sciences/Cancer HospitalAffiliated to, Shanxi Medical University, Taiyuan, People’s Republic of China

**Keywords:** Lung adenocarcinoma, Indeterminate pulmonary nodules, Piwi-interacting RNAs, Early detection, Nodules stratification, Machine learning

## Abstract

Early detection of lung adenocarcinoma (LUAD) remains a major clinical challenge despite the widespread application of low-dose computed tomography (LDCT). Circulating PIWI-interacting RNAs (piRNAs), characterized by tumor-specific expression and high stability, offer promise as non-invasive biomarkers. To improve the diagnostic precision of LDCT screening, we performed a large multi-center study integrating paired tissue–serum omics profiling with machine learning–based biomarker discovery. From 1,521 serum samples (1,033 LUAD, 89 benign pulmonary nodules, and 399 healthy controls), two tumor-derived PIWI-interacting RNAs (piR-hsa-8393202 and piR-hsa-8429916) were identified as highly stable, LUAD-specific molecules closely associated with disease progression. A 2-piRNA diagnostic signature demonstrated robust performance for early-stage LUAD (training AUC = 0.918; validation AUC = 0.863) and adenocarcinoma in situ (training AUC = 0.902; validation AUC = 0.907). Notably, when applied to LDCT-detected indeterminate pulmonary nodules, this signature significantly improved malignant nodule identification (AUC = 0.883), outperforming conventional serum biomarkers such as carcinoembryonic antigen and cytokeratin 19 fragment antigen 21–1. Functional assays further revealed that these piRNAs promote tumor cell proliferation and suppress apoptosis, supporting their oncogenic activity. Collectively, this study establishes circulating piRNAs as non-invasive and mechanistically relevant biomarkers for molecular stratification of pulmonary nodules within LDCT screening programs, providing a clinically applicable tool to refine early lung cancer diagnosis and guide individualized management.

**Supplementary Information:**

The online version contains supplementary material available at 10.1186/s43556-025-00368-2.

## Introduction

Lung adenocarcinoma (LUAD), the predominant histological subtype of non–small cell lung cancer (NSCLC), remains the leading cause of cancer-related mortality worldwide. According to *Cancer Statistics, 2025,* approximately 2.5 million new lung cancer cases and 1.9 million deaths are projected globally [1]. Despite significant progress in surgical management and targeted therapies, patient survival remains highly stage-dependent—exceeding 90% for stage IA but falling below 10% for stage IV—underscoring the urgent need for strategies enabling detection during the pre-metastatic window [2].

Low-dose computed tomography (LDCT) screening has reduced lung cancer mortality through early identification of pulmonary nodules in high-risk populations [3, 4]. However, its high false-positive rate often leads to unnecessary invasive procedures, radiation exposure, and patient anxiety. These limitations underscore the need for complementary molecular biomarkers to refine LDCT-based decision-making and improve nodule risk stratification [5, 6].

Circulating non-coding RNAs (ncRNAs) have emerged as promising minimally invasive biomarkers due to their stability, tissue specificity, and detectability in biofluids [7]. While microRNAs (miRNAs) and long non-coding RNAs (lncRNAs) have shown diagnostic and prognostic potential in lung cancer, PIWI-interacting RNAs (piRNAs)—a distinct class of small ncRNAs—are increasingly recognized for their regulatory roles in genome integrity, epigenetic modulation, and tumorigenesis [8]. Mounting evidence links aberrant piRNA expression with malignant phenotypes and poor prognosis across multiple cancer types, suggesting that circulating piRNAs may serve as a new layer of cancer-specific molecular indicators [9–11].

Here, we hypothesized that specific piRNAs are aberrantly expressed in LUAD and stably detectable in peripheral blood, enabling non-invasive detection of early-stage disease. To test this hypothesis, we integrated tissue–serum omics profiling with machine learning in large multicenter cohorts comprising LUAD, benign pulmonary nodules, and healthy individuals. We identified and validated a piRNA-based diagnostic model capable of accurately distinguishing malignant from benign nodules detected by LDCT. This work provides a clinically applicable framework for molecular stratification of pulmonary nodules and expands the diagnostic landscape of circulating non-coding RNAs in lung cancer.

## Results

### Participants and characteristics

A total of 1,653 participants were initially enrolled across all collaborating centers between 2021 and 2024, including histopathological confirmed LUAD patients, individuals with benign pulmonary nodules, and healthy controls. After applying predefined eligibility and quality-control criteria, 1,473 participants with 1,521 qualified serum samples were included in the final analyses, while 132 participants were excluded due to missing clinical information (*n* = 63), hemolyzed serum samples (*n* = 38), or lipemic and low-quality samples (*n* = 31). The overall enrollment, exclusion, and sample allocation procedures are summarized in Fig. S1.

**Fig. 1:**
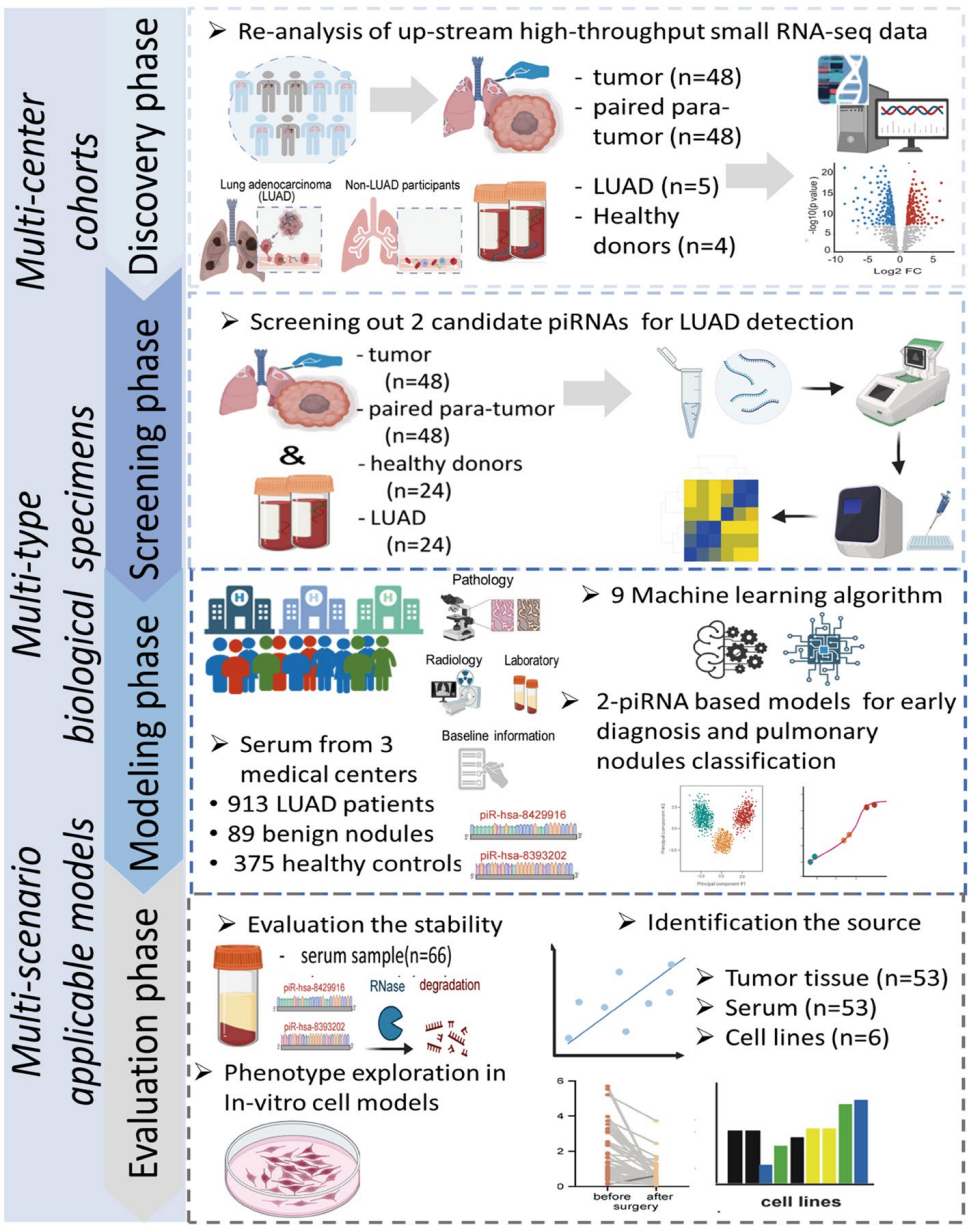
Overview of the workflow for the whole study. LUAD, lung adenocarcinoma. The illustration was created with a full license ID (CO28ZK0FBE) on BioRender.com

The study was designed in four sequential phases—discovery, screening, modeling, and evaluation (Fig. 1). In the discovery phase using public database, 48 paired LUAD tumor and adjacent normal tissues were analyzed, together with serum samples from 5 LUAD patients and 4 healthy donors. The screening phase included 48 paired LUAD and adjacent normal tissues, together with 24 LUAD and 24 healthy control serum samples for initial biomarker screening. The modeling and validation phase comprised 1,377 serum samples collected from three medical centers. The major cohort, retrospectively enrolled at the National Cancer Center (NCC, Beijing), included 711 LUAD cases, 72 benign nodules, and 245 healthy controls. The external validation cohort, established through multicenter collaboration during 2023–2024, consisted of 168 LUAD cases, 17 benign nodules, and 52 healthy controls from Henan Cancer Hospital, and an additional 34 LUAD cases and 78 healthy controls from Shanxi Provincial Cancer Hospital.

To verify the tumor-derived nature of the identified piRNAs, 48 LUAD patients with paired pre- and postoperative serum samples were further examined. The clinical characteristics of participants from the tissue and serum cohorts used in the discovery and screening phases are summarized in Table S1, and those from the modeling phase in Table S2.

In addition, a nested LDCT cohort of 350 participants with solitary pulmonary nodules (< 30 mm) was analyzed, encompassing pure ground-glass (*n* = 112), part-solid (*n* = 138), and solid (*n* = 100) nodules, with detailed radiologic–pathologic correlations provided in Table S3 [12].

### Discovery of candidate piRNAs simultaneously upregulated in serum samples and tumor tissues from patients with LUAD

**Fig. 2:**
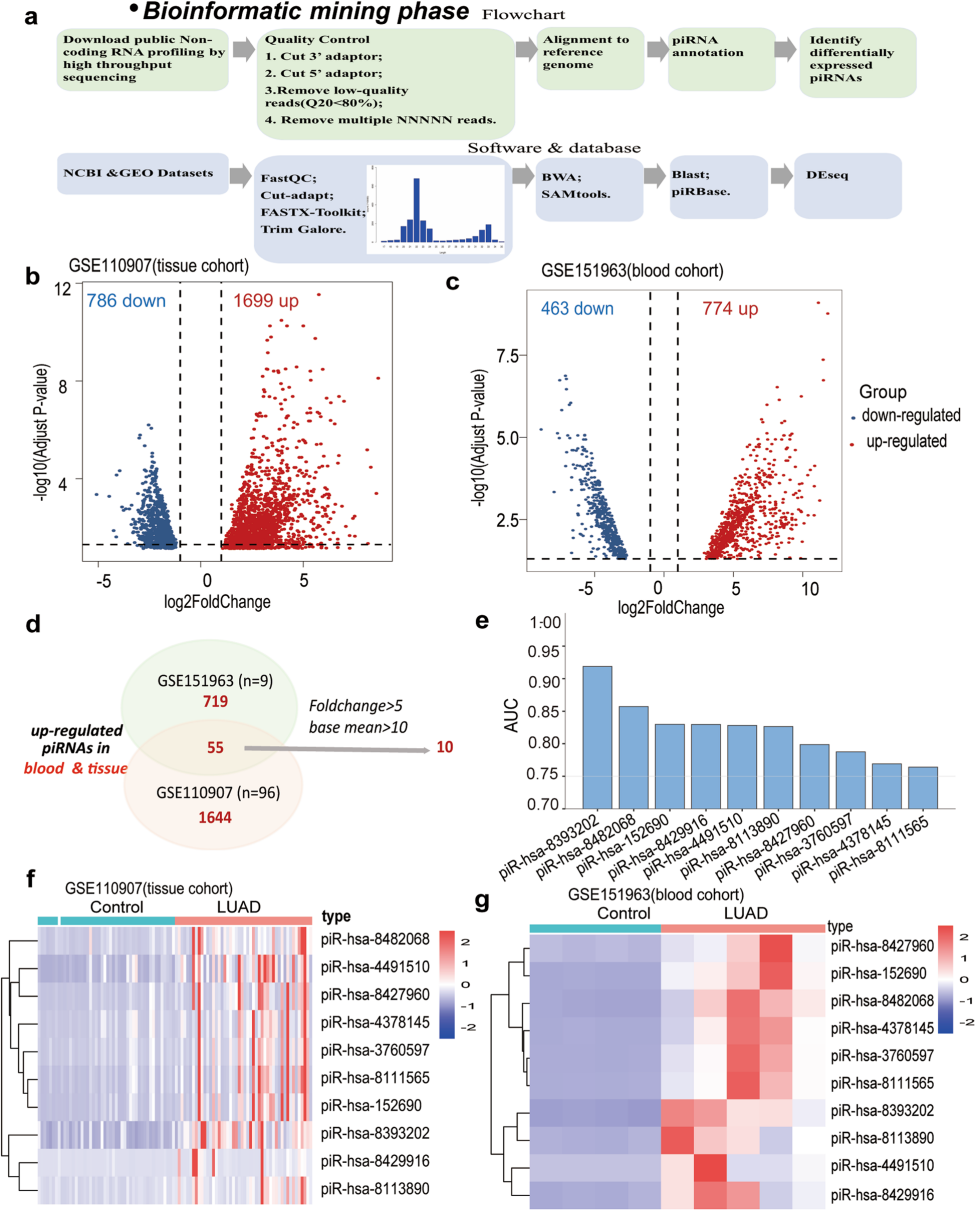
Discovering differentially expressed piRNAs in LUAD. **a** Workflow for the acquisition, expression profiling and differential analysis of piRNAs utilizing publicly available datasets. **b** Differentially expressed piRNAs between LUAD tumor tissues (*n* = 48) and paired adjacent noncancerous tissues (*n* = 48). **c**. Differentially expressed piRNAs between serum of LUAD patients (*n* = 4) and healthy donors (*n* = 5). **d**. Venn diagram of piRNAs which were simultaneously upregulated in serum samples and tumor tissues from LUAD patients. **e**. Top 10 candidate piRNAs along with their AUC values for distinguishing tumor tissues and paired adjacent normal tissues of LUAD. **f**, **g** Heatmap showing the expression of top 10 candidate piRNAs in LUAD tissue cohort (**f**) and serum cohort (**g**). LUAD, lung adenocarcinoma; AUC, area under the curve

To identify circulating piRNAs relevant to LUAD pathogenesis, we systematically reanalyzed publicly available small RNA sequencing datasets from the GEO database using a standardized bioinformatics workflow (Fig. 2a). Differential expression analysis, performed under stringent criteria (|log₂FC|> 1 and FDR < 0.05, Benjamini–Hochberg correction), revealed extensive piRNA dysregulation in both tumor tissues and serum samples.

In the tissue cohort, 1,699 piRNAs were significantly upregulated and 786 downregulated (Fig. 2b), whereas in the serum cohort, 774 were upregulated and 463 downregulated (Fig. 2c). To identify robust circulating candidates, we focused on piRNAs that were concurrently upregulated in both tissue and serum, yielding 55 shared molecules with consistent expression trends. These were further filtered by applying stringent thresholds (fold change > 5 and mean baseline expression > 10), narrowing the selection to ten candidate piRNAs with high diagnostic potential (Fig. 2d). Receiver operating characteristic (ROC) analysis confirmed their strong discriminative performance between LUAD and control samples (Fig. 2e). The expression patterns of these ten piRNAs were subsequently validated across independent tissue (Fig. 2f) and serum (Fig. 2g) cohorts, consistently showing significant upregulation in LUAD compared with normal controls. Together, these results identified a set of robust candidate piRNAs simultaneously upregulated in tumor tissues and circulation, providing a foundation for the development of non-invasive diagnostic biomarkers.

### Screening in the in-house cohort revealed 2 novel piRNAs for LUAD detection

**Fig. 3:**
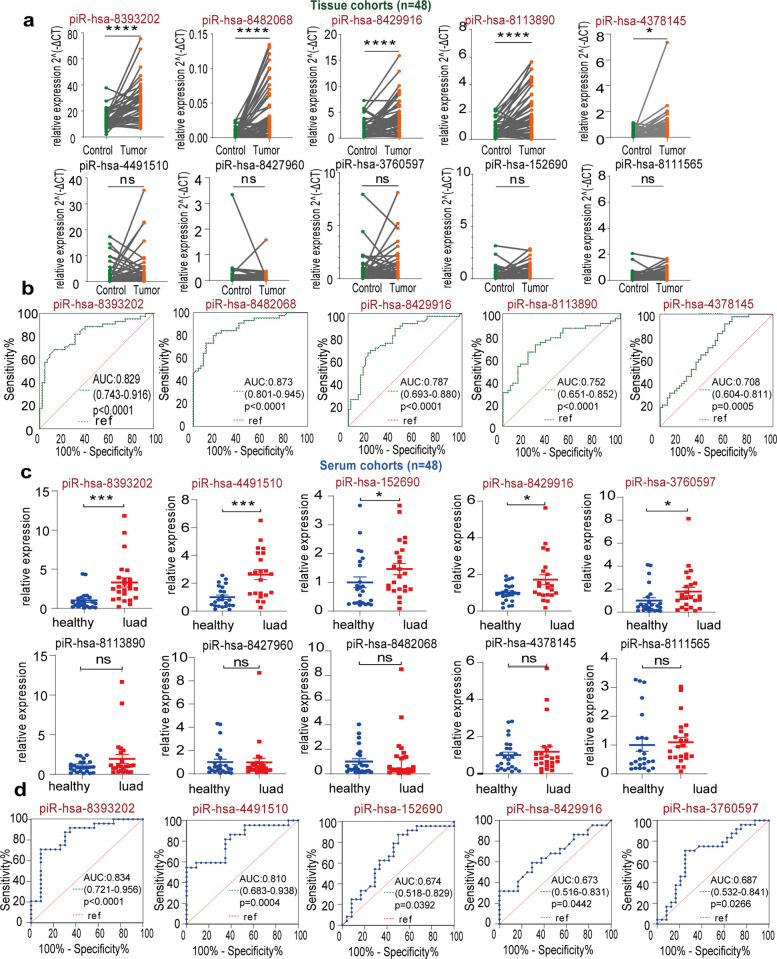
Screening out 10 candidate piRNAs in the tissue cohort and serum cohort. **a**. Expression levels of 10 candidate piRNAs in the tissue cohort (*n* = 48). Small nuclear RNA U6 was selected as the internal control for tissue sample quantification. **b**. ROC curves for candidate piRNAs in the tissue cohort, only piRNAs exhibiting differential expression through validation were presented exclusively. **c**. Expression levels of 10 candidate piRNAs in the serum cohort (*n* = 48). Hsa-miR-16-5p was selected as the internal control for serum sample quantification. **d**. ROC curves for candidate piRNAs in the serum cohort, only piRNAs exhibiting differential expression through validation were presented exclusively. Data are mean ± SEMs. *p < 0.05; **p < 0. 01; ***p < 0.001; ****p < 0.0001; ns *p* > 0.05. piRNAs, piwi-interacting RNAs; ROC, receiver operating characteristic

To validate the differential expression of the 10 prioritized piRNAs, we performed relative quantification in an independent local cohort. Clinical characteristics of enrolled participants are summarized in Table S1. Initial tissue-based analysis revealed significant upregulation of 5 piRNAs (piR-hsa-8393202, piR-hsa-8482068, piR-hsa-8429916, piR-hsa-8113890, piR-hsa-4378145) in LUAD tumors compared with matched adjacent normal tissues (Fig. 3a). They demonstrated promising discriminative capacity with AUC values of 0.829 (95% CI: 0.743–0.916), 0.873 (0.801–0.945), 0.787 (0.693–0.880), 0.752 (0.651–0.852), and 0.708 (0.604–0.811) respectively (Fig. 3b). Subsequent serum profiling identified a distinct set of 5 upregulated piRNAs (piR-hsa-8393202, piR-hsa-4491510, piR-hsa-8429916, piR-hsa-8113890, piR-hsa-4378145) in LUAD patients versus healthy controls (Mann–Whitney U test; Fig. 3c), exhibiting diagnostic AUCs of 0.834 (0.721–0.956), 0.810 (0.683–0.938), 0.674 (0.518–0.829), 0.673 (0.516–0.831), and 0.687 (0.532–0.841) (Fig. 3d).

Notably, only piR-hsa-8393202 and piR-hsa-8429916 displayed consistent upregulation across both tumor tissues and serum samples. This cross-compartmental elevation underscores their potential as specific biomarkers for LUAD diagnosis.

### Model construction based on piR-hsa-8393202 and piR-hsa-8429916 for LUAD diagnosis

**Fig. 4:**
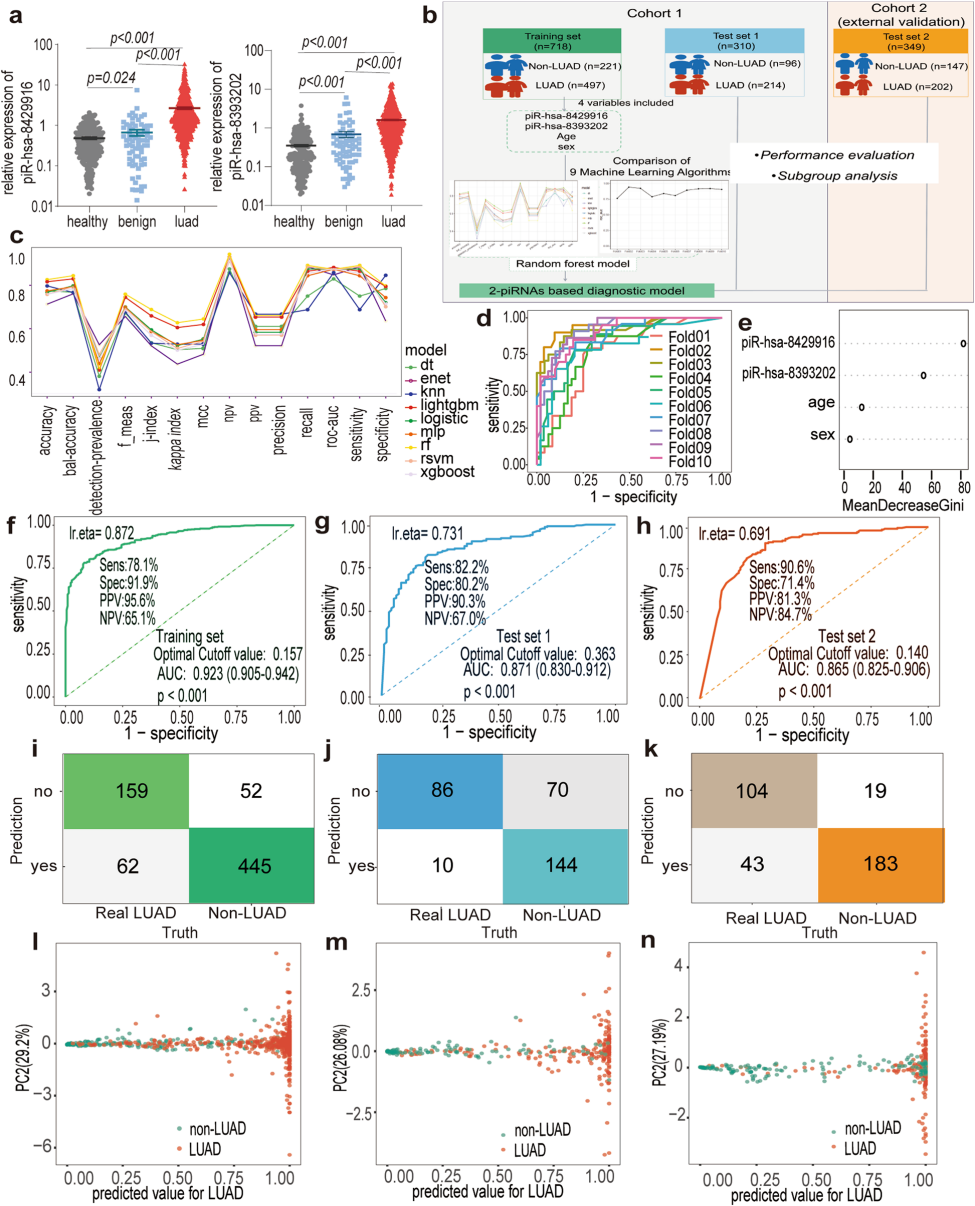
Modeling a 2-piRNAs based signature for LUAD diagnosis. **a**. The expression levels of the piR-hsa-8429916 (left) and piR-hsa-8393202 (right) in the healthy (*n* = 711), benign (*n* = 72) and LUAD (*n* = 245) subgroups. Data are mean ± SEMs. **b**. Workflow showing the development of diagnostic model. **c**. Comparative evaluation of diagnostic performance metrics of nine distinct machine learning paradigms. **d**. ROC curves for each fold of the tenfold cross-validation by random forest. **e**. Beeswarm plot illustrating the impact of each feature on the model outcomes by Shapely values. **f**, **g**, **h** The ROC curves for the diagnosis of LUAD patients in the training set (**f**), test set 1 (**g**), test set 2 (**h**). **i**, **j**, **k** Confusion tables of binary results of the diagnostic model in the training set (**i**), test set 1 (**j**), test set 2 (**k**). **l**, **m**, **n** PCA plot showing the prediction performance of the diagnostic model for distinguishing LUAD (colored in red) from non-LUAD (colored in green) in the training set (**l**), test set 1 (**m**), test set 2 (**n**). piRNA, piwi-interacting RNA; LUAD, lung adenocarcinoma; ROC, receiver operating characteristic; PCA, Principal component analysis

To establish a robust diagnostic model based on the two most promising piRNAs, piR-hsa-8393202 and piR-hsa-8429916, their serum expression levels were quantified in 711 LUAD patients, 72 individuals with benign pulmonary diseases (BPD), and 245 healthy controls (HC) from the National Cancer Center (Fig. 4a). Both piRNAs showed markedly elevated expression in LUAD patients compared with the BPD and HC groups (all *p* < 0.001), and intergroup analysis further revealed significant differences between HCs and BPDs, confirming their potential clinical relevance. For model construction and validation, all participants were randomly divided into an independent training cohort (*n* = 718; 497 LUAD and 221 non-LUAD) and a non-overlapping test cohort (*n* = 310; 214 LUAD and 96 non-LUAD), with age and sex evenly distributed between groups (*p* > 0.05; Fig. 4b). Notably, early-stage disease predominated, with stage 0–II LUADs accounting for 89.5% (445/497) in the training and 86.9% (186/214) in the test cohort (Table S2).

Nine machine-learning algorithms, including logistic regression, support vector machine, random forest, gradient boosting, k-nearest neighbors, naïve Bayes, neural network, elastic net, and decision tree, were systematically compared to identify the optimal predictive approach. ROC analysis demonstrated that the random forest model achieved the highest classification accuracy (0.826) and superior AUC performance relative to alternative algorithms (range, 0.773–0.816; *p* < 0.01; Fig. 4c). Accordingly, the random forest classifier was selected for subsequent analyses due to its balanced precision-recall performance (F1-score = 0.891). Ten-fold cross-validation further confirmed its robustness, yielding a mean AUC of 0.869 ± 0.02 (range: 0.762–0.941) with narrow confidence intervals (95% CI: 0.823–0.915) and low variance (Fig. 4d), indicating strong generalizability and minimal overfitting.

Model interpretability analysis using SHapley Additive exPlanations (SHAP) revealed that piR-hsa-8429916 contributed most significantly to LUAD discrimination (SHAP values: 0.2–0.6), followed by piR-hsa-8393202 (− 0.2 to 0.4), while age and sex had negligible influence (Fig. 4e). Collectively, these results establish a two-piRNA-based random forest model with high accuracy, reproducibility, and interpretability for distinguishing LUAD from benign and healthy cases.

### Model validation for LUAD diagnosis in multi-center retrospective cohorts

The diagnostic model demonstrated robust discriminative capacity for LUAD detection across multicenter cohorts. In the training set (Fig. 4f), the ROC analysis yielded an AUC of 0.923 (95% CI: 0.905–0.942; *P* < 0.001), with sensitivity of 78.1%, specificity of 91.9%, PPV of 92.5%, and NPV of 55.6% at the optimal cutoff value of 0.157. Internal validation in Test set 1 (Fig. 4g) achieved an AUC of 0.871 (95% CI: 0.830–0.912; *P* < 0.001) with 82.2% sensitivity, 82.4% specificity, 93.5% PPV, and 57.3% NPV (cutoff = 0.363). External validation Test set 2 (Fig. 4h) exhibited comparable performance (AUC = 0.865; 95% CI: 0.825–0.906; *P* < 0.001), demonstrating 90.8% sensitivity, 71.4% specificity, 81.4% PPV, and 84.7% NPV (cutoff = 0.140).

Classification accuracy was further quantified through confusion matrix analysis (Fig. 4i-k). The training set correctly identified 445 LUAD cases (true positives) and 159 non-LUAD controls (true negatives), with 62 false-negative and 52 false-positive classifications. Test set 1 showed 86 true positives, 144 true negatives, 10 false negatives, and 10 false positives, while Test set 2 contained 43 true positives, 183 true negatives, 19 false positives, and 104 false negatives. PCA plots (Fig. 4l-n) confirmed the model's discriminatory power, with distinct clustering of high predicted-value LUAD cases versus low predicted-value non-LUAD controls along the primary variance component 1(PC1), supporting the model's biological interpretability. Besides, subgroup analysis of the 2-piRNA diagnostic panel across different histological grades (Grade 1–3) was performed (Table 1). The 2-piRNA signature maintained robust diagnostic accuracy in well-differentiated LUAD (Grade 1) with an AUC of 0.915 (95% CI: 0.906–0.922, *p* < 0.001), while the diagnostic performance remained statistically significant in Grade 2 (AUC = 0.876, *p* < 0.001) and Grade 3 tumors (AUC = 0.830, *p* = 0.004).

**Table 1:**
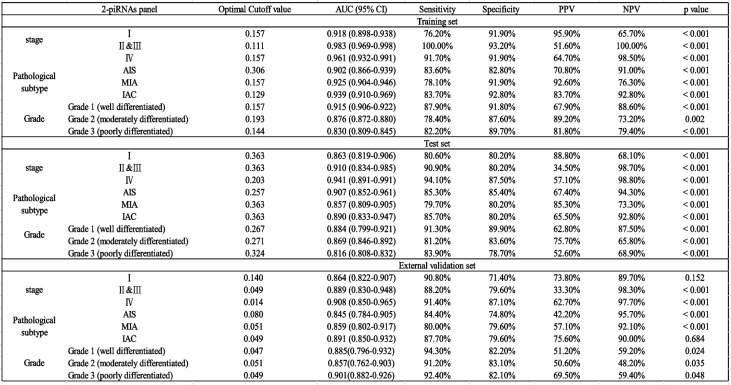
Subgroup performance of 2-piRNAs based model for LUAD diagnosis

Collectively, these results suggest that the 2-piRNA panel performs consistently across LUAD tumors of varying differentiation degrees, especially in detecting early and well-differentiated lesions with malignant potential.

### Model construction based on piR-hsa-8393202 and piR-hsa-8429916 for Indetermined Pulmonary Nodule Classification

**Fig. 5:**
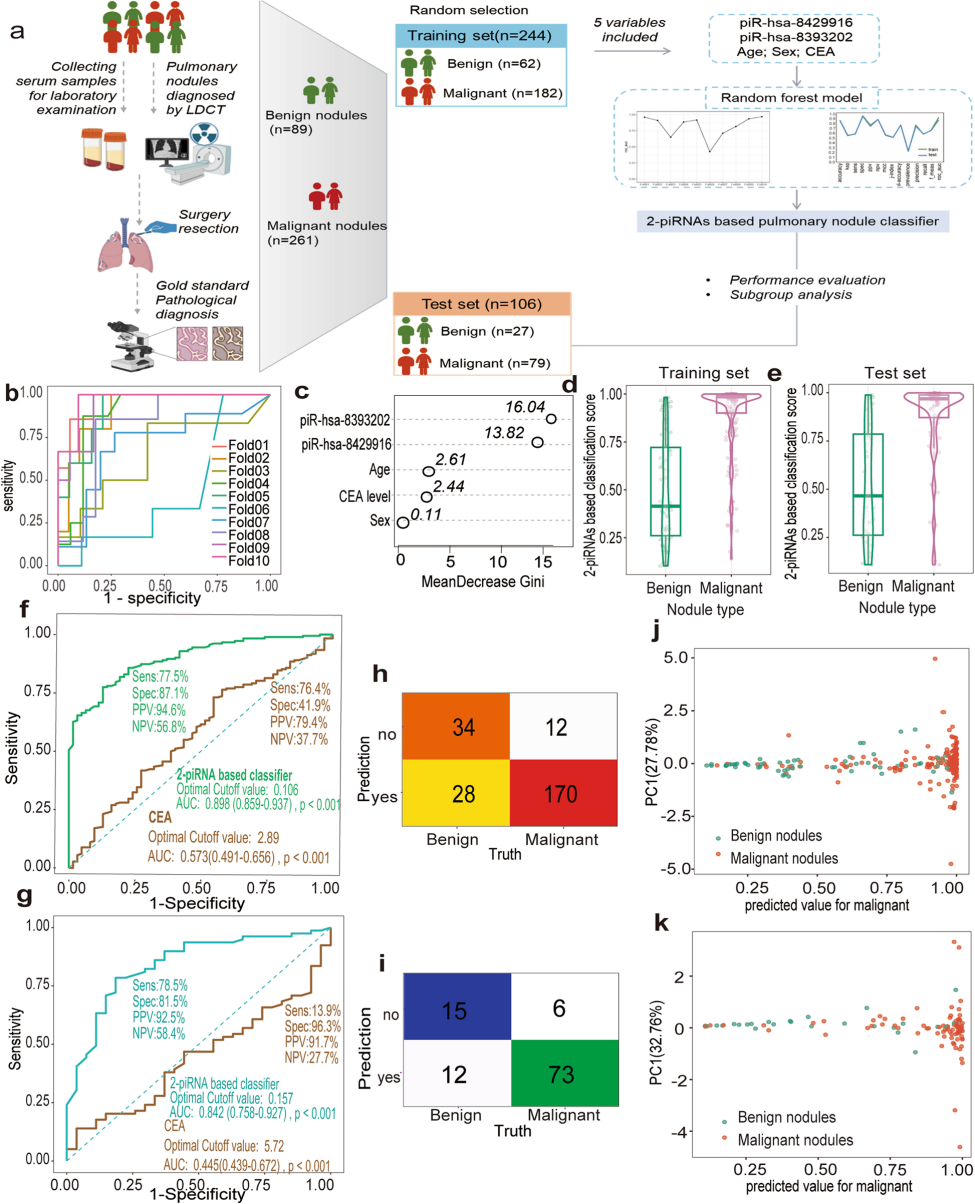
Modeling a 2-piRNAs based model for pulmonary nodule classification. **a**. Schematic depicting the development of 2-piRNAs based pulmonary nodule classifier. The illustration was created with a full license id (IK28ZK02S5) on BioRender.com. **b**. ROC curves for each fold of the tenfold cross-validation by random forest. **c**. Relative variable importance evaluated by mean decrease Gini index, higher values indicate a greater role. **d**, **e** 2-piRNAs based classifier score in patients with benign and malignant nodules in training set (**d**) and test set (**e**). The box element delineates interquartile range. **f**, **g** The ROC curves of 2-piRNAs based classifier and CEA for pulmonary nodule classification in training set (**f**) and test set (**g**). **h**, **i** Confusion tables of binary results of the pulmonary nodule classification model in the training set (**h**) and test set (**i**). **j**, **k** PCA plot showing the prediction performance of the classification model for distinguishing malignant nodules (colored in red) from benign nodules (colored in green) in the training set (**j**), test set (**k**). piRNAs, piwi-interacting RNAs; ROC, receiver operating characteristic; CEA, Carcinoembryonic antigen; PCA, Principal component analysis

We aimed to assess the capacity of the 2-piRNA classifier to effectively stratify indeterminate pulmonary nodules (IPNs). A total of 350 samples exhibiting pulmonary nodules on LDCT images were selected from the study participants (Fig. 5a; detailed demographic characteristics are provided in Table S3). A random forest algorithm was developed, utilizing 2 piRNAs (piR-hsa-8429916 and piR-hsa-8393202), alongside age, sex, and carcinoembryonic antigen (CEA) levels. This model was built using a training cohort comprising 244 samples (62 benign and 182 malignant) and independently validated with an additional cohort of 106 samples (27 benign and 79 malignant). The stability of the model was evaluated through tenfold cross-validation on the training cohort, which exhibited consistent ROC curve trajectories across all iterations (Fig. 5b). The aggregated AUC was found to be 0.885 (95% CI: 0.863–0.907), indicating robust discriminatory capacity and a negligible tendency for overfitting. MeanDecrease Gini analysis highlighted both piRNA markers as the primary distinguishing features, demonstrating significantly higher variable importance compared to demographic or conventional biomarkers (Fig. 5c). Box plot distributions indicated notable score variations between benign and malignant groups, both in the training set (Fig. 5d) and the test set (Fig. 5e). The 2-piRNA classifier exhibited superior diagnostic efficacy across both cohorts, with ROC analysis yielding an AUC of 0.885 (95% CI: 0.859–0.927), achieving 77.5% sensitivity and 84.1% specificity in the training cohort, significantly surpassing CEA, which had an AUC of 0.573 (Fig. 5f). External validation demonstrated sustained high performance (AUC = 0.842, 95% CI: 0.786–0.897; sensitivity = 78.5%, specificity = 82.4%), showing marked improvement over CEA (AUC = 0.445; Fig. 5g). Additionally, confusion matrix analysis further supported the diagnostic precision for both benign and malignant subclasses (Fig. 5h, i), underscoring the classifier's clinical utility in the stratification of pulmonary nodules. PCA visualization revealed distinct spatial separation of pathologically confirmed benign (green) and malignant (red) nodules (Fig. 5j, k).

### Model validation and subgroup analysis for Indetermined Pulmonary Nodule Classification

**Fig. 6:**
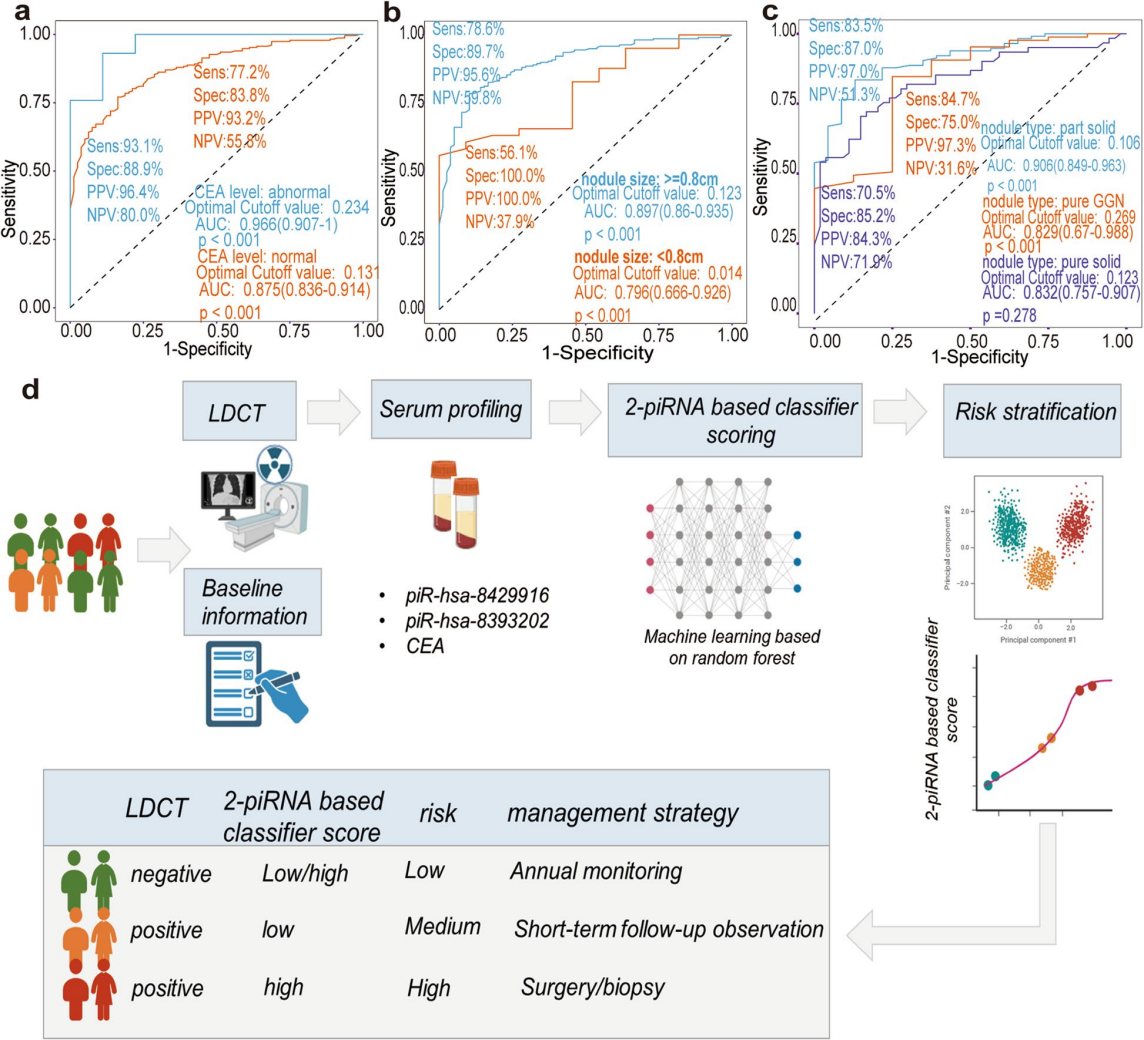
Subgroup performance of 2-piRNAs based pulmonary nodule classifier. **a**. ROC curve of 2-piRNAs based classifier among different CEA status. **b**. ROC curve of 2-piRNAs based classifier in different nodule sizes. **c**. ROC curve of 2-piRNAs based classifier in different nodule radiological subtypes. **d**. The risk stratification process for classifying pulmonary nodules based on 2-piRNA based classifiers. The illustration was created with a full license id (LW28ZK0QQ9) on BioRender.com. piRNAs, piwi-interacting RNAs; ROC, receiver operating characteristic; CEA, Carcinoembryonic antigen; GCN, ground-glass nodule

Further investigation was undertaken to evaluate the diagnostic performance of the 2-piRNA based pulmonary nodule classifier across various subgroups. In the subgroup stratified by CEA levels (Fig. 6a), the group with elevated CEA levels achieved an impressive AUC of 0.966, accompanied by a sensitivity of 93.1% and a specificity of 88.9%. This finding underscores a markedly higher diagnostic efficiency relative to the CEA-normal group, which exhibited an AUC of 0.875. When stratified by nodule size (Fig. 6b), patients with nodules measuring ≥ 0.8 cm demonstrated an AUC of 0.897, with sensitivity at 78.6% and specificity at 89.7%. In contrast, the cohort with nodules smaller than 0.8 cm exhibited a lower diagnostic performance, as indicated by an AUC of 0.796. When analyzing the subgroup categorized by nodule type (Fig. 6c), the part-solid nodule group attained the highest AUC of 0.906, with a sensitivity of 83.5% and specificity of 87.0%. The pure ground-glass nodule group yielded an AUC of 0.829, while the pure solid nodule group recorded an AUC of 0.832, highlighting the variability in diagnostic capability among different nodule types. CEA and CYFRA 21–1 are the most well-studied laboratory markers in the context of pulmonary nodule classification [13–15]. To further evaluate their diagnostic performance, we performed a comparative analysis between the 2-piRNA based classifier and established laboratory markers. Importantly, the classifier consistently outperformed CEA and CYFRA 21–1 testing in distinguishing benign from malignant pulmonary nodules across all subgroup analyses (Table S4). Collectively, the non-invasive detection of 2 piRNAs and LDCT can reduce the false positives of LDCT and accurately stratify the treatment for LUAD (Fig. 6d).

### Tumor-derived piR-hsa-8393202 and piR-hsa-8429916 exhibit high serum stability and pro-oncogenic functions in LUAD

**Fig. 7:**
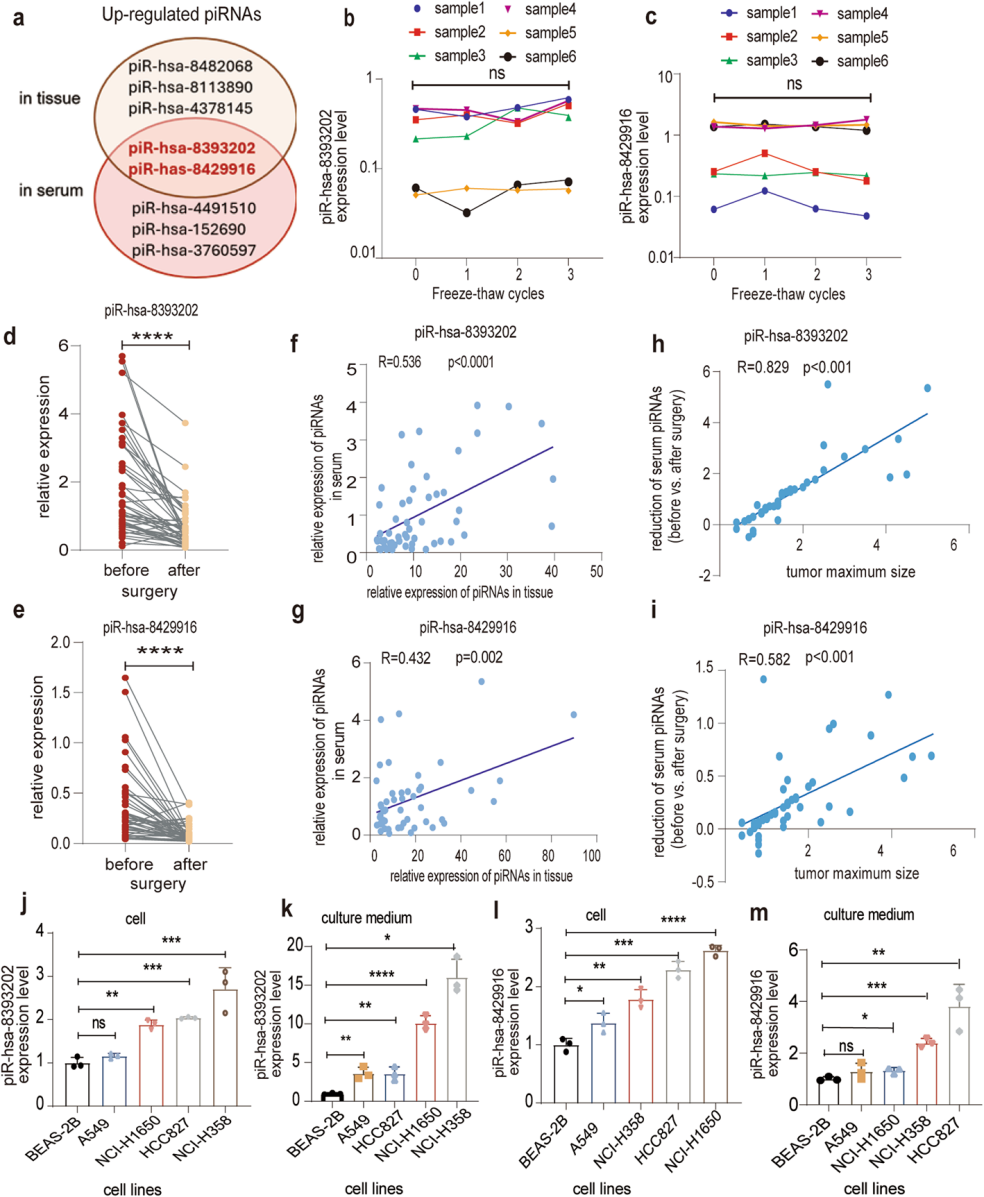
Evaluation of the stability and source of piR-hsa-8429916 and piR-has-8393202. **a**. Venn diagram of upregulated piRNAs in the tumor tissue and LUAD serum cohorts. **b**, **c** Expression of piR-hsa-8393202 (**b**) and piR-hsa-8429916 (**c**) in serum samples undergoing repeated freeze–thaw cycles. **d**, **e** Comparison of serum piR-hsa-8393202 (**d**) and piR-hsa-8429916 (**e**) in paired before and after surgery samples (*n* = 48). **f**, **g** Correlations of the relative expression of piR-hsa-8393202 (**f**) and piR-hsa-8429916 (**g**) between paired serum and tissue samples (*n* = 45). **h**, **i** Correlations between the reduction of serum piR-hsa-8393202 (**h**) and piR-hsa-8429916 (**i**) expression and tumor maximum size (*n* = 53). **j**, **k** piR-hsa-8393202 levels in both cells (**j**) and culture media (**k**) of normal lung epithelial cell line BEAS-2B and some LUAD cell lines. **l**, **m** piR-hsa-8429916 levels in both cells (**l**) and culture media (**m**) of normal lung epithelial cell line BEAS-2B and some LUAD cell lines. Data are mean ± SEMs. *p < 0.05; **p < 0. 01; ***p < 0.001; ****p < 0.0001; ns, *p* > 0.05. piRNAs, piwi-interacting RNAs; LUAD, lung adenocarcinoma

We next characterized the biological origin, molecular stability, and functional relevance of the two piRNAs in LUAD. Both piR-hsa-8393202 and piR-hsa-8429916 exhibited remarkable stability in serum specimens subjected to repeated freeze–thaw cycles, maintaining consistent expression levels across three cycles as measured by RT-qPCR (Fig. 7a–c). To determine their tumor-derived source, paired pre- and postoperative serum samples from 48 LUAD patients were analyzed. Circulating levels of both piRNAs significantly decreased after tumor resection (*p* < 0.001 for each; Fig. 7d, e), and their serum expression showed positive correlations with matched tumor tissue levels (piR-hsa-8393202: R = 0.536, *p* < 0.001; piR-hsa-8429916: R = 0.432, *p* = 0.002; Fig. 7f, g). Moreover, the extent of postoperative reduction correlated with tumor volume (piR-hsa-8393202: R = 0.829; piR-hsa-8429916: R = 582; *p* < 0.001 for both; Fig. 7h and i), further supporting their tumor-derived origin.

Cell culture experiments confirmed tumor cell-specific expression and secretion. Adherent cells (2*10^6) were cultured in 10 cm medium for 12 h before the collection of cells and cell-free culture medium. Both piRNAs were significantly overexpressed in LUAD cell lines (Fig. 7j, l) in contrast with normal bronchial epithelial cell line (BEAS-2B, *n* = 3). Notably, conditioned media from LUAD cells contained significantly higher levels of piR-hsa-8393202 and piR-hsa-8429916 compared to BEAS-2B cell supernatants (Fig. 7k, m), indicating active secretion by tumor cells.

Given their tumor-specific elevation in both tissues and circulation, we next explored their biological functions in LUAD cells. Two LUAD cell lines, NCI-H358 and HCC827, which displayed high endogenous expression of both piRNAs, were transfected with sequence-specific inhibitors. Efficient transfection was confirmed by RT-qPCR (Fig. S2a, S2b). Functional assays demonstrated that inhibition of either piRNA significantly suppressed tumor cell growth, as shown by CCK-8 assays (Figures S2c, S2d). Flow cytometry analysis further revealed a marked increase in Annexin V/PI double-positive cells upon piRNA knockdown, indicating enhanced apoptosis (Fig. S2e, S2f). Together, these findings establish piR-hsa-8393202 and piR-hsa-8429916 as stable, tumor-derived circulating piRNAs that exert pro-oncogenic effects in LUAD, promoting tumor cell proliferation and survival while maintaining excellent molecular stability suitable for clinical biomarker application.

## Discussion

This multicenter study identifies piR-hsa-8393202 and piR-hsa-8429916 as novel, tumor-derived circulating biomarkers for the early detection of LUAD and molecular classification of IPNs. Through integrative analysis of paired tissue–serum omics data and multi-institutional validation in 1,653 participants, we demonstrate that these two piRNAs exhibit the core attributes of ideal liquid biopsy biomarkers: tumor specificity, molecular stability, and early diagnostic capability. Their significant reduction after surgical resection and correlation with tumor volume strongly support a tumor-derived origin, whereas their stability across multiple freeze–thaw cycles underscores analytical robustness. Consistent upregulation in both LUAD tissues and matched serum suggests active secretion via exosomal or vesicular pathways, which was further supported by in vitro evidence of piRNA enrichment in conditioned media from LUAD cell [16]. These observations align with emerging paradigms of small RNA–mediated intercellular communication within the tumor microenvironment.

Our findings extend current understanding of non-coding RNA biology in LUAD. While microRNAs and long non-coding RNAs have been widely studied as diagnostic biomarkers, the functional and translational relevance of piRNAs remains largely unexplored. Previous studies have implicated piRNAs in cancer stemness, genomic stability, and immune modulation, yet their clinical detectability and mechanistic contribution in LUAD were unclear [17]. The present study bridges this gap by establishing two specific circulating piRNAs that not only discriminate LUAD from benign lesions but also promote tumor proliferation and inhibit apoptosis in vitro, suggesting a dual role as both biomarkers and functional mediators of tumor progression. These findings reveal a potentially novel oncogenic mechanism linked to piRNA dysregulation in lung cancer.

Despite its strengths, this study has several limitations. First, the retrospective design may introduce selection bias, as all LUAD cases were confined to treatment-naïve patients undergoing surgical resection. Prospective, multicenter studies with longitudinal follow-up in LDCT-positive populations are needed to validate the real-world clinical utility of the two-piRNA signature for monitoring malignant transformation. Second, although our mechanistic experiments indicate that these piRNAs exert pro-oncogenic effects, the molecular underpinnings—particularly their biogenesis, exosomal packaging, and interaction with Argonaute or PIWI proteins—remain to be elucidated. Finally, interation of piRNA profiles with radiomic features or proteomic markers may further enhance diagnostic precision, warranting future integrative biomarker studies.

In summary, this study establishes piR-hsa-8393202 and piR-hsa-8429916 as robust, tumor-derived, and functionally relevant circulating biomarkers for LUAD detection and IPN stratification. The two-piRNA signature exhibits consistent diagnostic performance across independent cohorts (AUC > 0.84), analytical stability, and superior accuracy compared with conventional biomarkers. By addressing long-standing challenges in the specificity and reproducibility of liquid biopsy, our findings highlight the translational potential of circulating piRNAs as a molecular adjunct to LDCT, offering a foundation for personalized and biologically informed management of pulmonary nodules.

## Methods

### Study design

A prospective-specimen collection, retrospective blinded-evaluation (PRoBE) cohort study was conducted from 2021–2024 [18]. A total of 1,653 participants was enrolled from 3 medical centers in 3 independent provinces and municipalities in China. A total of 1,653 individuals were initially enrolled across all participating centers. Following quality control and eligibility screening, 1473 participants with 1521 qualified serum samples and 48 paired LUAD and adjacent normal tissues were retained for downstream analyses. The study protocol was approved by the institutional review boards of the National Cancer Center (NCC2021C-527), conforming to the Declaration of Helsinki (2013 revision). Written informed consent was obtained from all participants prior to enrollment. This multi-center study followed the TRIPOD guideline for transparent reporting of diagnostic prediction models (EQUATOR Network) [19]. The completed TRIPOD reporting checklist is provided in Supplementary File.

### Participants enrollment

All LUAD diagnoses adhered to the WHO Classification of Thoracic Tumors (5th edition) and were staged using the IASLC 8th edition TNM system [20, 21]. LUAD patients were treatment-naïve, with blood collected prior to biopsy, surgery, or systemic therapy. Exclusion criteria included: (1) concurrent malignancies; (2) prior chemotherapy/immunotherapy; (3) severe hepatic/renal dysfunction (AST/ALT > 3*ULN, eGFR < 30 mL/min/1.73m^2^); (4) active infections (HIV, HBV) or autoimmune diseases; (5) age below 18 years. Controls were age-, sex-, and smoking status-matched to cases, with normal inflammatory markers (CRP < 5 mg/L, leukocyte count 4–10 × 10⁹/L).

All patient staging was determined according to the 8th edition of the AJCC/UICC TNM classification, which was the clinical standard at the time of study design (2021–2024). Both the GEO datasets used for biomarker discovery (GSE110907, GSE151963) and the institutional validation cohort were based on the same edition. The 9th edition, effective January 2025, mainly refines N and M descriptors for advanced disease, which does not affect the early-stage LUAD analyses in this study.

Benign nodule cases were confirmed via histopathology, excluding individuals with familial cancer history or occupational carcinogen exposure. The pathological types of benign pulmonary nodules include bronchial adenoma, chronic granulomatous lesions, atypical adenomatous hyperplasia, and conditions such as chronic obstructive pulmonary disease. Subjects underwent at least three consecutive CT scans. Tumor size was defined as the maximum diameter of the solid component on thin-section CT, in accordance with the IASLC 8th edition TNM classification. For part-solid nodules, only the solid portion was measured. SUVmax values were obtained from PET-CT reports when available and summarized in Supplementary Table S3.

Healthy controls underwent comprehensive screening, including tumor marker assays (CEA, AFP), chest X-ray, and abdominal/pelvic ultrasound, to exclude occult malignancies or inflammatory conditions.

### Public data mining and candidate piRNAs screening

Small RNA sequencing data were obtained from LUAD tumor tissues and serum samples, accessed through the Gene Expression Omnibus (GEO) under accession numbers GSE110907 and GSE151963. Raw sequencing reads were converted to FASTQ format using the SRA Toolkit (v3.0.0) (https://www.ncbi.nlm.nih.gov/sra/docs/toolkitsoft/) and subjected to quality assessment with FastQC. Reads meeting the following criteria were retained: (1) length distribution compatible with piRNA biology (26–32 nt), (2) base call quality ≥ Q30 in > 80% of cycles, and (3) absence of adapter contamination.

Adapter trimming and quality filtering were performed using Cutadapt (v3.4) with parameters: *-q 20 –minimum-length 18 –maximum-length 35 –trim-n*. piRNA annotation files (piRBase v2.0) were mapped to the human reference genome (GRCh38) using BWA (v0.7.17) with stringent alignment parameters: *-n 1 -k 11 -l 11 (allowing* ≤ *1 mismatch)* [22]. Aligned reads were processed with SAMtools (v1.12) to generate sorted BAM files [23].

Read counts were computed utilizing HTseq-count (version 0.9.1) in union-counting mode [24]. Piwi-interacting RNAs (piRNAs) exhibiting counts per million (CPM) ≥ 1 in at least 20% of tumor samples were preserved for subsequent analysis. Data normalization was conducted employing the trimmed mean of M-values (TMM) method. Differentially expressed piRNAs were discerned using DESeq2 (v1.34.0) [25], applying thresholds of |log2(fold change)|≥ 1 and a false discovery rate (FDR) of less than 0.05, as determined by the Benjamini–Hochberg correction.

### Collection of tissue specimens

Forty-eight pairs of LUAD and matched adjacent normal tissue samples were collected. Samples were obtained from patients who had been diagnosed with primary LUAD by pathological examination of tissue biopsy and had surgical resection operations at the Cancer Hospital of the Chinese Academy of Medical Sciences (Beijing, China) from September 2021 to March 2022. The clinical information for all patients enrolled is listed in Table S1. Tissue specimens were immediately stored in liquid nitrogen after resection.

### Serum collection and processing

Peripheral venous blood (3–4 mL) was drawn after a 12-h fasting period using serum vacutainer tubes. Samples were centrifuged at 1,500 × g for 10 min within 30 min of collection to minimize hemolysis. Serum aliquots were stored at − 80 °C until analysis, with rigorous quality control: samples exhibiting hemolysis (absorbance > 0.3 at 414 nm) or improper storage were excluded.

### Total RNA isolation and RT-qPCR

Total RNA from clinical tissue specimens was isolated using TRIzol Reagent (Invitrogen, Cat# 15,596,018), while serum-derived RNA was extracted with TRIzol LS (Invitrogen, Cat# 10,296,010) to optimize recovery of low-abundance RNAs. Reverse transcription was conducted utilizing a poly(A)-tailing-based PrimeScript™ RT Master Mix (Sangon Biotech, Catalog No. B532451), in accordance with the manufacturer’s protocols.

Quantitative PCR analysis was conducted using a Roche LightCycler® 480 II system, incorporating SYBR Green detection chemistry (Roche, Catalog No. 04887352001). The primer sequences utilized for piRNA amplification are detailed in Supplementary Table S5. Tissue RNA quantification was normalized to U6 snRNA, while serum samples included exogenous cel-miR-39 spike-in controls (Thermo Fisher, Catalog No. 4395408) in addition to endogenous miR-16-5p for data normalization [26]. Three independent biological replicates were analyzed for each sample. Relative expression was calculated using the comparative ΔΔCt method, with melt curve analysis confirming reaction specificity [27].

### Cell lines and culture

The human normal alveolar epithelial cell line BEAS-2B and LUAD cell lines (A549, NCI-H1650, HCC827, and NCI-H358) were obtained from the Cell Bank of the Shanghai Institute of Biochemistry and Cell Biology, Chinese Academy of Sciences (Shanghai, China). BEAS-2B cells were sourced from the China National Cell Culture Center (Beijing, China). All cell lines were maintained for fewer than six passages post-thawing and authenticated through short tandem repeat (STR) profiling. Mycoplasma contamination was routinely excluded using PCR-based detection. BEAS-2B cells were cultured in Dulbecco’s Modified Eagle Medium (DMEM; Gibco, Grand Island, NY, USA), supplemented with 10% fetal bovine serum (FBS; Gibco). A549 cells were maintained in DMEM containing 10% FBS and 100 U/mL penicillin/streptomycin (Gibco). NCI-H1650, HCC827, and NCI-H358 cells were grown in RPMI-1640 medium (Gibco), with 10% FBS and antibiotics. All cells were incubated at 37 °C in a humidified atmosphere with 5% CO₂. For piRNA analysis, 1 × 10⁶ cells were seeded in 10-cm dishes and allowed to adhere overnight. The cells were then serum-starved in FBS-free medium for 4 h. Both cellular pellets and the corresponding cell-free culture supernatants were collected for subsequent quantification of piR-hsa-8429916 and piR-hsa-8393202.

### Construction of the diagnostic model

The diagnostic prediction model was developed using the Tidymodels package (v0.1.4) in R (v4.3.0). Participants in Cohort 1 (*n* = 1,028) were divided into a training set (*n* = 718) and an internal test set (*n* = 310) via stratified random sampling, with an external validation cohort (*n* = 349) from independent centers included for generalizability assessment [28]. A modular workflow was implemented for nine machine learning algorithms (logistic regression, support vector machine, random forest, gradient boosting, k-nearest neighbors, naive Bayes, neural network, elastic net, and decision tree). All models were trained using 4 predictive variables: expression levels of 2 piRNAs, age, and gender. Data preprocessing included normalization (z-score for continuous variables) and class imbalance correction via SMOTE [29].

For the optimized random forest model, 1,000 decision trees were generated through dual randomization: bootstrap resampling (80% sample replacement) and feature subspace selection. Hyperparameter tuning employed Latin hypercube grid search across 20 combinations of mtry (2–4) and min_n (5–20), evaluated by stratified tenfold cross-validation. Model performance was assessed using receiver operating characteristic (ROC)-AUC (primary metric), sensitivity, specificity, positive predictive value (PPV), and negative predictive value (NPV) [30, 31]. Permutation importance analysis identified key predictors, with computational reproducibility ensured through fixed random seeds and parallel processing (8-core CPU).

Model explainability was achieved via SHAP (SHapley Additive exPlanations) values, calculated using Monte Carlo approximation with 50 iterations and bias correction [32]. Analyses were implemented via the “fastshap” R package, ensuring additive consistency and clinical interpretability.

ROC curves were generated using the multipleROC package, with DeLong's test comparing AUC differences between models [33, 34].

### Construction of the classifier model

A cohort of 350 participants with IPNs was analyzed to develop a binary classification model using five predictive variables. The random forest algorithm was implemented via the tidymodels framework (v0.1.4) in R (v4.3.0), following Transparent Reporting of a Multivariable Prediction Model for Individual Prognosis or Diagnosis (TRIPOD) guidelines [34]. Stratified random sampling allocated 75% (*n* = 262) to training and 25% (*n* = 88) to testing. Missing values (< 2% of data) were imputed using median replacement, followed by z-score normalization. Hyperparameter optimization employed a grid search across m try (2–4) and min_n (5–20) combinations, evaluated through tenfold stratified cross-validation. The final random forest ensemble comprised 1,000 decision trees, leveraging bootstrap aggregation and feature subspace randomization to enhance generalizability.

Model selection prioritized ROC-AUC while balancing sensitivity and specificity trade-offs. Diagnostic thresholds were determined via Youden index maximization, with bootstrapped confidence intervals (1,000 iterations) quantifying metric stability. Performance evaluation was conducted on the test sets. The “multiROC” package was utilized to plot the combined ROC curve. Feature importance was quantified via Gini impurity-based MeanDecreaseGini and visualized using gradient-colored plots from the “vip” package. A multi-level random seed strategy ensured reproducibility across data partitioning, model training, and validation. principal component analysis (PCA) was performed on z-score normalized features using singular value decomposition, retaining components explaining > 95% cumulative variance.

### Statistical analysis

A two-sided Wilcoxon rank-sum test was used when comparing two groups for unpaired samples. The Wilcoxon signed-rank test was employed for nonparametric analysis of paired samples. A two-sided Kruskal–Wallis test was used when comparing three or more groups. The Spearman rank correlation (rho) was applied to analysis correlation between two variables, and associated *p*-value was calculated by two-tailed test. The area under the receiver operating characteristic curve (AUROC), precision–recall (PR) curve, sensitivity, specificity, positive predictive value (PPV), negative predictive value (NPV), and overall accuracy were used to evaluate the diagnostic performance. The DeLong test was used to evaluate the significance for the difference of AUCs. Data in abnormal distributions were analyzed by nonparametric tests. All statistical analyses were performed using SPSS 20.0 (IBM), GraphPad Prism (v.9.0) and R (v.3.6.0) software (https://www.r-project.org/). *P* < 0.05 was considered statistically significant.

## Supplementary Information

Supplementary Material 1.

## Data Availability

All data supporting this study are available within the paper and its Supplementary Information.
