# Supplementary material for: Identification of PIWI-interacting RNAs based models for lung adenocarcinoma early detection: a multicenter cohort study
Source: Mol Biomed. 2025 Nov 30;6:127. doi: 10.1186/s43556-025-00368-2 (PMC12665640; doi:10.1186/s43556-025-00368-2)
Supplement: Supplementary file 1 — Supplementary Material 1. [file 43556_2025_368_MOESM1_ESM.docx]

**Title page**

**Title:** Identification of piwi-interacting RNAs Based Models for Lung Adenocarcinoma Early Detection: A Multicenter Cohort Study.

**Authors:** Shuang Liang ^1, 1+^, Qian Hong ^2,3, 1+^, Qingxia Xu ^4,^ ^1+^, Yuan Wang ^1^, Yue Wu ^1^, Juwei Mu ^2^, Chunyan Wang ^5^, Hezhi Fang ^1^*, Wei Cui ^1,4^*.

^1^ Department of Clinical Laboratory, State Key Laboratory of Molecular Oncology, National Cancer Center/National Clinical Research Center for Cancer/Cancer Hospital, Chinese Academy of Medical Sciences and Peking Union Medical College, Beijing 100021, China

^2^ Department of Thoracic Surgery, National Cancer Center/National Clinical Research Center for Cancer/Cancer Hospital, Chinese Academy of Medical Sciences and Peking Union Medical College, Beijing 100021, China.

^3^ Department of Thoracic Surgery, Sir Run Run Shaw Hospital, School of Medicine, Zhejiang University, Hangzhou, Zhejiang 310058, China.

^4^ Department of Clinical laboratory, Affiliated Cancer Hospital of Zhengzhou University & Henan Cancer Hospital, Zhengzhou, Henan, China.

^5^ Department of Transfusion, Shanxi Province Cancer Hospital/Shanxi Hospital Affiliated to Cancer Hospital, Chinese Academy of Medical Sciences/Cancer Hospital Affiliated to Shanxi Medical University, Taiyuan, People's Republic of China.

^1+^ Shuang Liang, Qian Hong and Qingxia Xu contributed equally to this work and are regarded as co-first authors.

*Correspondence: [cui123@cicams.ac.cn](mailto:cui123@cicams.ac.cn) (Wei Cui); [fangh@cicams.ac.cn](mailto:fangh@cicams.ac.cn) (Hezhi Fang)

Supplementary fig 1. Flowchart detailing the sources of participants and the exclusion criteria.


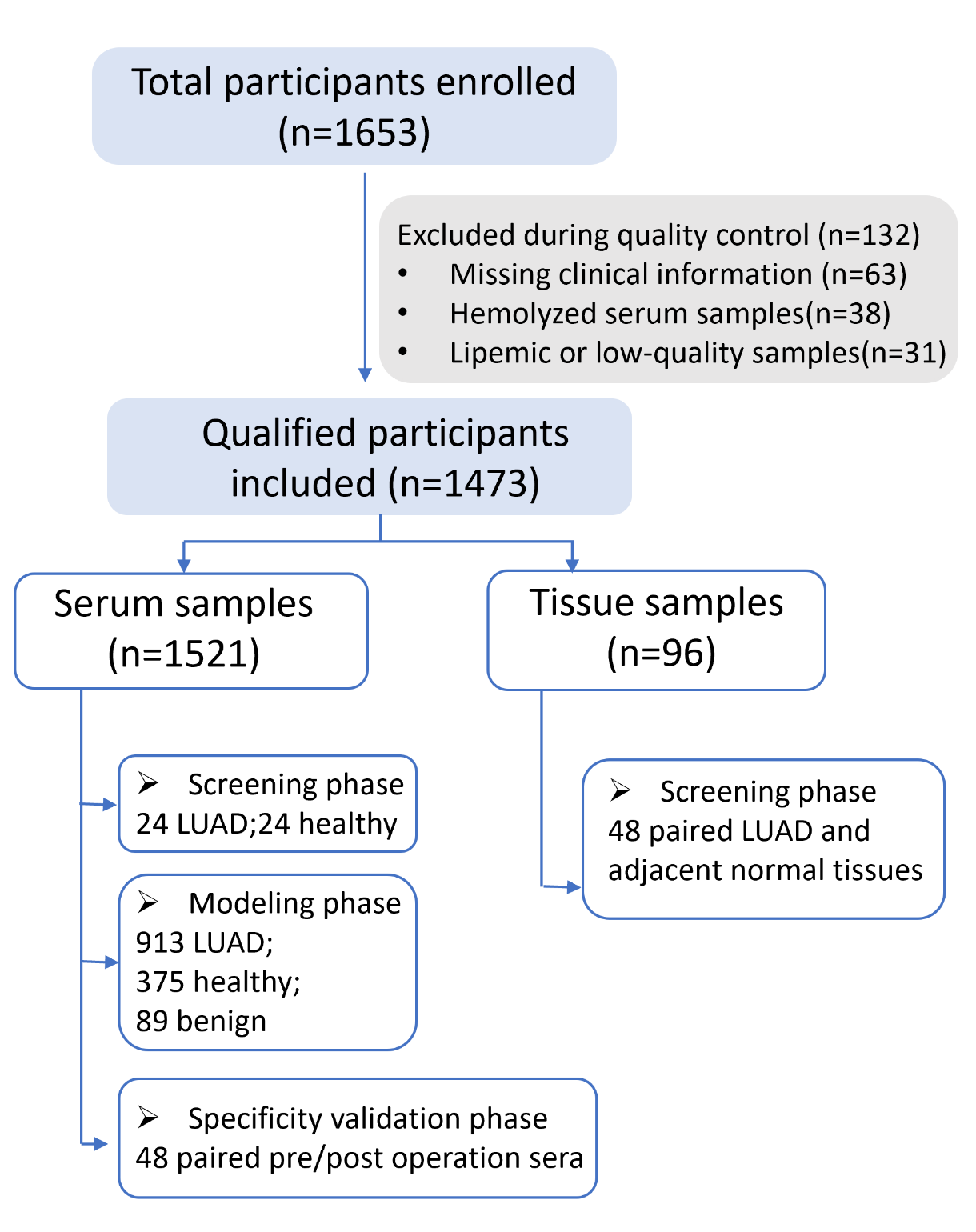


Supplementary fig 2. Suppressing 2 piRNAs inhibit the growth of lung cancer tumor cells.

**a, b** RT-qPCR confirmed piRNA inhibitors down-regulated the level of piR-hsa-8393202 (a) and piR-hsa-8429916 (b) in cells.

**c, d** Cell proliferation curves of HCC827 (c) and NCI-H358 (d) cells transfected with piR-hsa-8393202 inhibitor and piR-hsa-8429916 inhibitor were detected by CCK-8 assay. OD values at 450 nm were measured daily for 5 days.

**e, f** Representative images of colony formation assay in HCC827 (e) and NCI-H358 (f) cells after transfection with piR-hsa-8393202 inhibitor and piR-hsa-8429916 inhibitor. Colonies were stained and visualized.

**g, h** Apoptosis analysis of HCC827 and NCI-H358 cells after 2 piRNAs inhibition. (g) Representative flow cytometry plots for Annexin V/PI staining; (h) Quantification of apoptotic cell percentages.

Data are presented as mean ± SD. ns, p>0.05; * P < 0.05, ** P < 0.01, ***P < 0.001, ****P < 0.0001

CCK-8, Cell counting kit 8


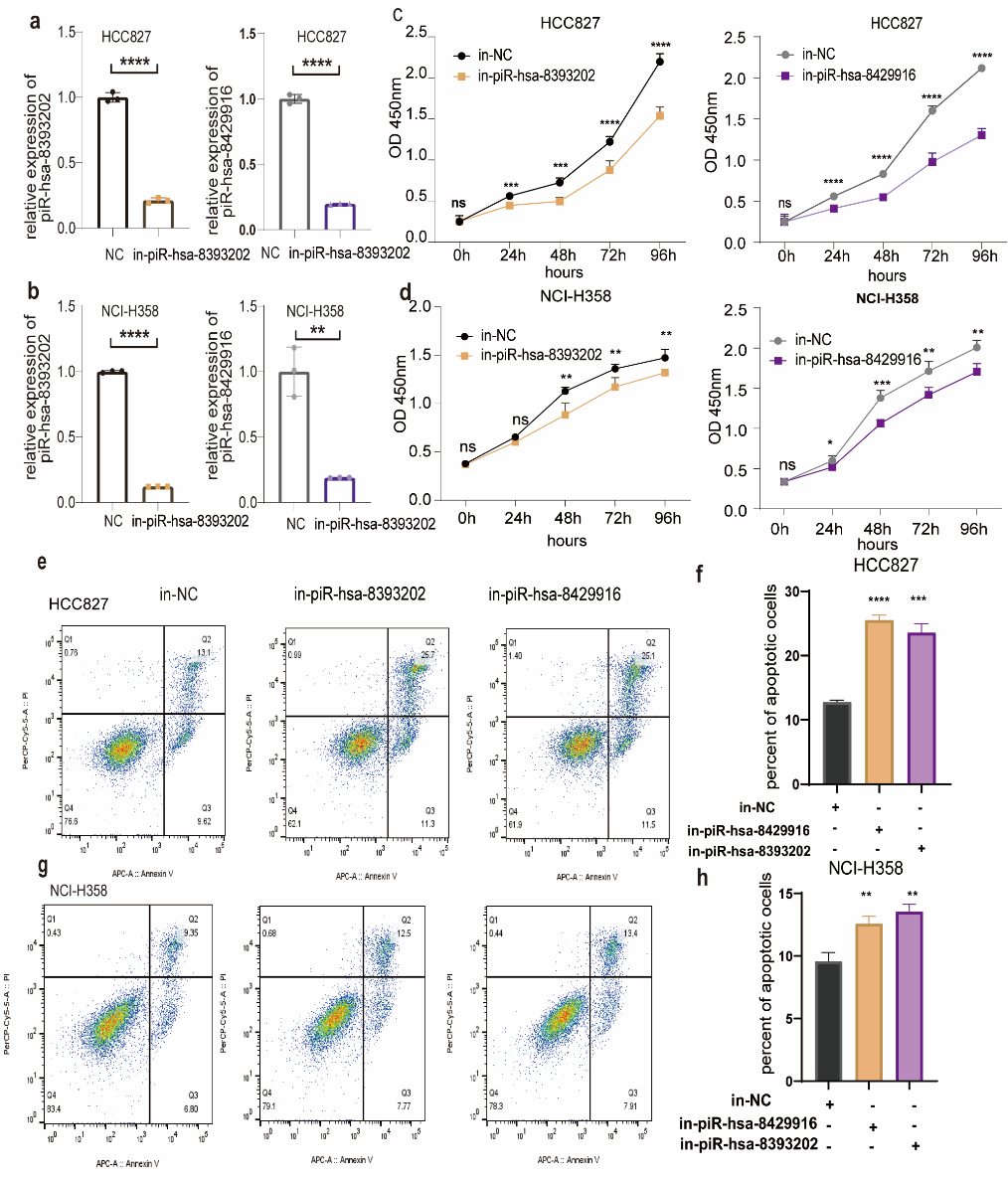


Table.S1 The clinical information of participants from the LUAD tissue cohort and serum cohorts in screening phase.


Table.S2: clinical characteristics of the enrolled participants for modeling phase

Table S.3: clinical characteristics of the enrolled participants with Indeterminate Pulmonary Nodules

Table. S4. Subgroup performance of 2-piRNAs based classifier, serum CEA level and CYFRA21-1 level for IPN classification.


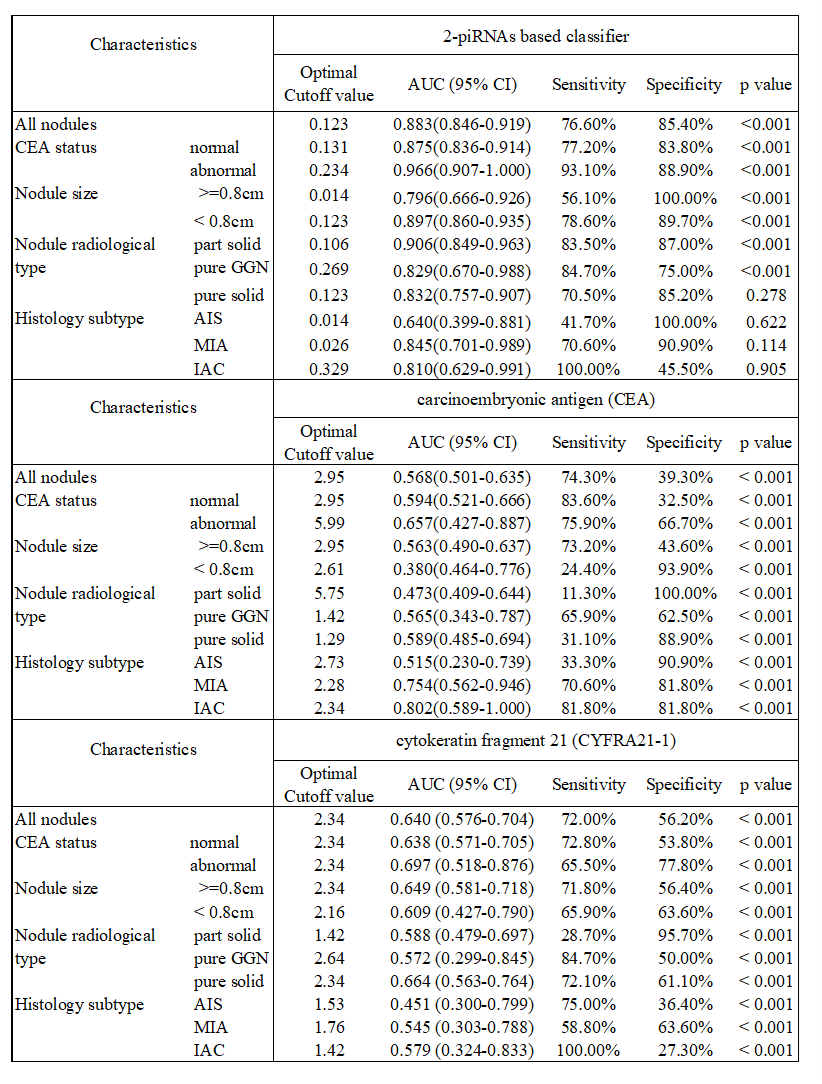


Table.S5 The primer sequences utilized for piRNA validation.

TRIPOD Checklist of this study


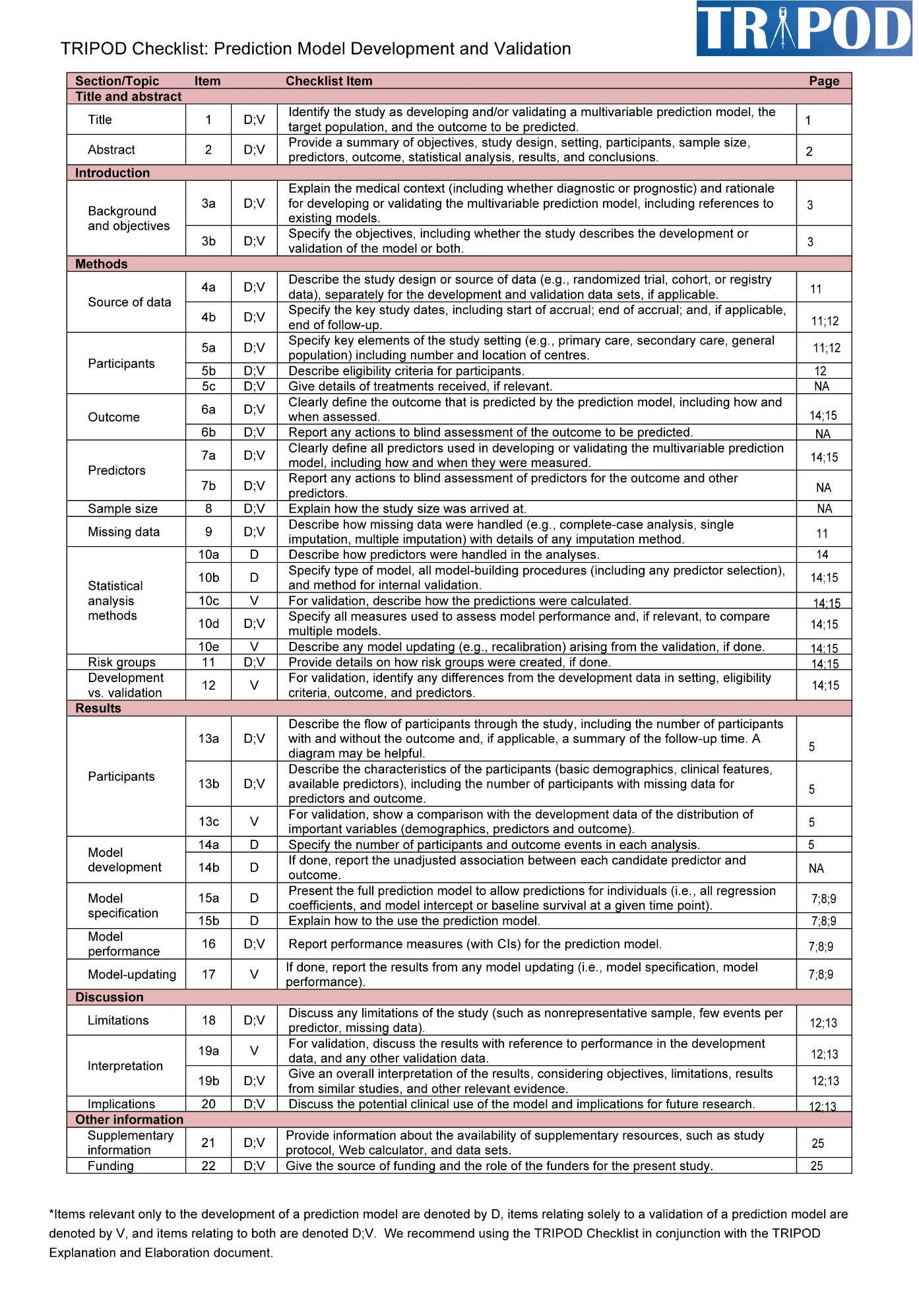


Methods

1. **inhibitor transfection**

For piRNA inhibition, cells wer e seeded in 6-well plates and transfected with piRNA inhibitors (in-piR-hsa-8393202: GCGACCGCAGUCCCCCACUACCACAAA, in-piR-hsa-8429916: ACCCCCUGCUCCAAAAAUCCAUUUAAUA, Genepharma) or negative control (in-NC) using Lipofectamine™ 3000 (Invitrogen) following the manufacturer’s protocol. Relative expression levels of piRNAs were calculated using the 2^(-ΔΔCt) method, with U6 snRNA as the internal control.

1. **CCK-8 Assay for Cell Proliferation**

Transfected cells were seeded into 96-well plates (3×10^3 cells/well). At 0h, 24h, 48h, 72h, and 96h post-seeding, 10μL of cell counting kit 8 (CCK-8) (Dojindo) was added to each well. After incubating at 37 °C for 1.5h, the optical density (OD) at 450 nm was measured using a microplate reader.

1. **Flow Cytometry for Apoptosis Detection**

Transfected cells were harvested, washed and resuspended in 1×binding buffer. Then, 5μL of Annexin V-APC and 5μL of propidium iodide (PI) were added. After incubating in the dark for 15 min, cells were analyzed using a flow cytometer. Apoptotic cells were quantified as the percentage Annexin V and PI double positive cells.

1. **Statistical analysis**

A two-sided Wilcoxon rank-sum test was used when comparing two groups for unpaired samples. The Wilcoxon signed-rank test was employed for nonparametric analysis of paired samples. Data in abnormal distributions were analyzed by nonparametric tests. All statistical analyses were performed using SPSS 20.0 (IBM), GraphPad Prism (v.9.0) and R (v.3.6.0) software. P<0.05 was considered statistically significant.
